# Association of Posterior Tibial Slope and Functional Outcome After Total Knee Arthroplasty: An Observational Study

**DOI:** 10.7759/cureus.74201

**Published:** 2024-11-22

**Authors:** Arpit Singh, Prakhar Verma, Narendra S Kushwaha, Ravindra Mohan, Devarshi Rastogi

**Affiliations:** 1 Department of Orthopaedic Surgery, King George's Medical University, Lucknow, IND

**Keywords:** knee osteoarthritis (koa), knee range of motion (rom), kujala score, pts (posterior tibial score), tka (total knee arthroplasty)

## Abstract

Introduction

Precise implant positioning, particularly a well-balanced posterior tibial slope (PTS), is crucial for the success of total knee arthroplasty (TKA) because it enhances sagittal plane stability and significantly influences knee motion patterns. The long-term impact of tibial slope on active and passive range of motion (ROM) still needs to be studied, despite ROM's crucial role in patient contentment. This study examined the relationship between tibial slope and active and passive ROM following TKA, with a follow-up period of at least three months.

Materials and methods

The study included 40 knees of 30 participants who had undergone initial TKA for knee osteoarthritis (KOA). These subjects were recruited between September 2023 and May 2024, with a minimum postoperative follow-up period of three months.

Results

Significant improvements in the average Kujala score and knee ROM were observed at postop day 7 and at the three-month follow-up as compared to preoperative measurements (p<0.001). Concurrently, there was a notable decrease in the mean posterior tibial slope (PTS) angle (p<0.001). While patients with a larger PTS angle exhibited higher average Kujala scores and knee ROM, the correlations between these variables and the PTS angle were not statistically significant.

Conclusion

Considering its limitations, this research indicates that the posterior tibial slope does not significantly affect the functional outcomes of patients after primary total knee replacement in the initial treatment phase.

## Introduction

Total knee arthroplasty (TKA) is a common surgical intervention for patients with end-stage degenerative knee arthritis experiencing persistent pain and restricted joint movement. It is aimed at relieving pain and recovering the natural knee range of motion (ROM) by restoring normal mobility and stability at the replaced joint. The extent of movement after TKA is affected by various biomechanical factors. These include the angle of the posterior tibial slope (PTS), the specific design features of the implant, the offset of the posterior femoral condyle (PFCO), how well the soft tissues are balanced, and the size of the prosthesis used to replace the articulating surfaces of the knee joint [1]. Malalignment in any of these biomechanical factors causes mechanical imbalance and patellofemoral complications with early failure due to polyethylene wear and stability problems. The alteration of tibial inclination substantially impacts the knee's kinematics [2-4]. A balanced PTS is crucial for maintaining stability in the sagittal plane. The tibial slope is a significant factor; it is generally believed that an increased posterior tibial slope can greatly improve the postoperative maximal flexion of the knee. However, this assumption is not substantiated in literature reviews.

The PTS is characterized by the angle between the line perpendicular to the mid-diaphysis and the tangent to the medial or lateral tibial plateau [5]. The tibial slope affects numerous aspects of knee functionality. These include the knee's stability, maximum bending capacity, resting position, cruciate ligament tension, cartilage pressure, and implant stress in TKA cases. On average, human knees exhibit a slope of about 8°. While no notable differences exist between males and females, variations have been noted amongst different ethnic groups [6-14].

A crucial factor in assessing the efficacy of TKA is the ROM. Enhanced mobility correlates with better functionality and higher patient contentment. Various studies have shown that individuals typically require 67° of flexion for the gait swing phase, 83° for ascending stairs, 90° for descending stairs, and 93° to stand up from a seated position [15-19].

In TKA, a steeper posterior slope may contribute to an enhanced ROM. In vitro studies indicate a correlation between the tibial cutting angle and the resulting flexion. Specifically, prostheses fitted with a 0° tibial slope demonstrated notably reduced ROM compared to knees cut with increased slopes of 4° or 7° [6,12]. Despite ROM being a crucial factor in patients' overall satisfaction, the impact of tibial slope on long-term outcomes, particularly regarding active and passive ROM, has not been thoroughly investigated. Our study sought to examine the potential relationship between tibial slope and ROM following TKA, with a minimum follow-up period of three months.

## Materials and methods

Study design and setting

This prospective observational study included 40 knees of 30 patients (16 females and 14 males) with knee osteoarthritis (KOA) who have undergone primary TKA (posterior stabilized) at the Department of Orthopaedic Surgery, King George Medical University UP, Lucknow during the study period of April 2023 to April 2024. This study was approved by the Institutional Ethics Committee, KGMU (XIX-PGTSC-IIA/P42).

Inclusion and exclusion criteria

**Figure 1:**
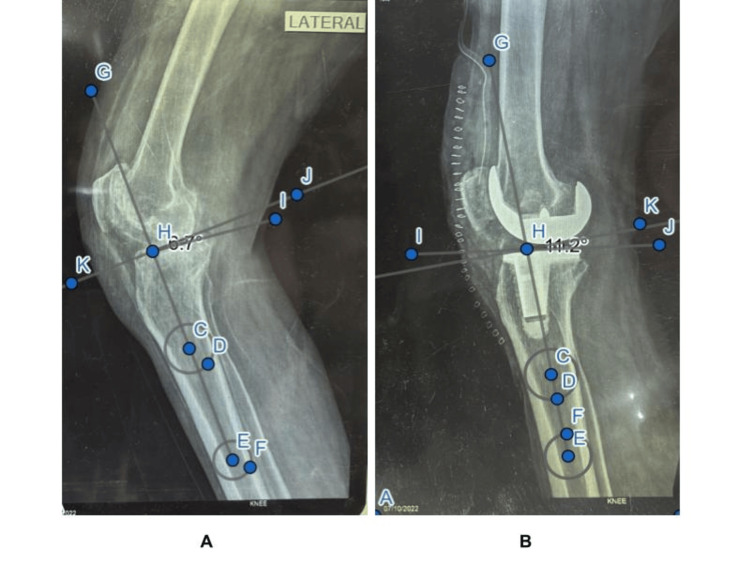
A 65-year-old male admitted with bilateral KOA (right > left), managed by TKA of the right knee A. Preoperative; B. Postoperative KOA: knee osteoarthritis; TKA: total knee arthroplasty

Patients treated for KOA with primary TKA and with at least three months of follow-up were included in the study (Figure 1). Patients with secondary KOA, including those with valgus deformities, previous knee surgery, fracture sequelae, and neurological disorder associations, as well as individuals who have undergone revision surgery or experienced complications during the follow-up period, have been excluded from the study.

Data collection

Demographic information, such as age, gender, place of residence, history of smoking, education level, and history of arthritis, was collected from the participants after getting their consent. The completed three-month rehabilitation was assessed by taking a medical history, checking the general condition, and examining the knee radiologically with a true lateral long leg X-ray, along with other relevant investigations pertaining to our study (Figure 1).

**Figure 2:**
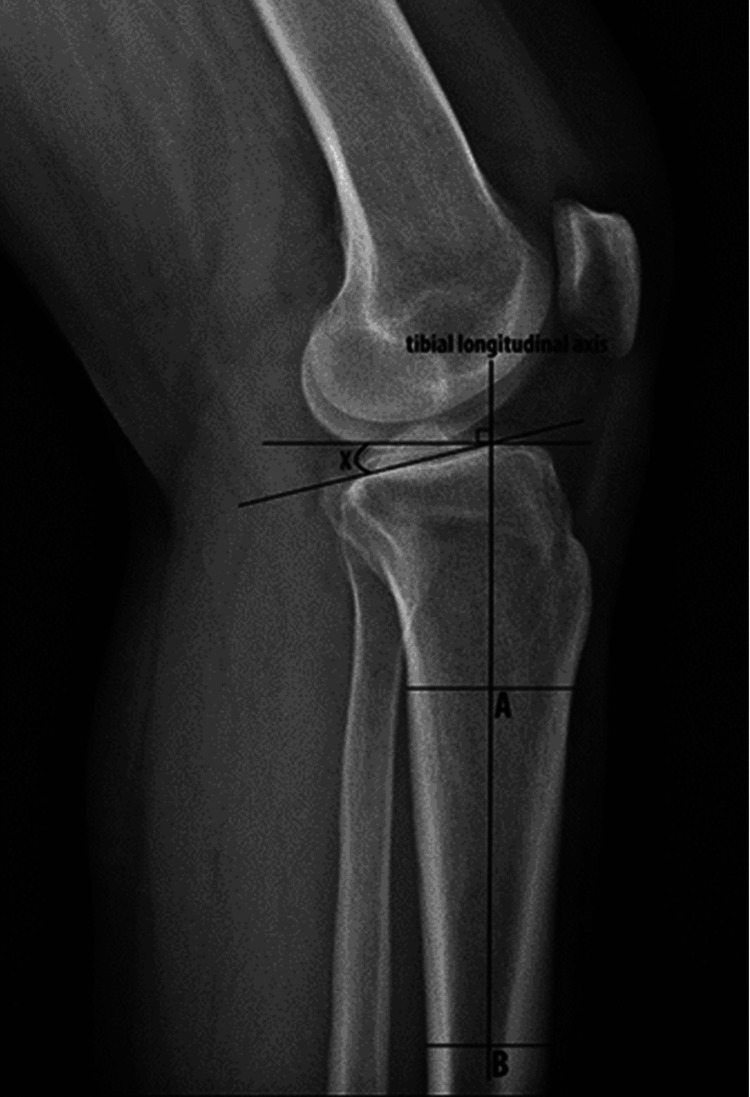
Posterior tibial slope

On the lateral X-ray of the knee, two parallel lines are drawn perpendicular to the tibial cortex at diaphysis. Then, a line is drawn connecting the mid-point of these two parallel lines, extending up to the knee joint. A perpendicular line is drawn to the mid-diaphyseal line and another tangent line is drawn to the medial tibial plateau passing through the mid-diaphyseal line. Then, the angle between these two lines is measured as the PTS angle (Figure 2) [5].

Statistical analysis

The SPSS version 24.0 software (IBM Corp., Armonk, NY, US) was employed for conducting statistical analyses. Continuous variables were described using mean (standard deviation) or range values as appropriate. To assess the significance of differences in means, the student's t-test was utilized for comparisons between the two groups. Changes in continuous variables within a group were evaluated using a paired t-test. Spearman correlation was used to examine the relationship between two continuous variables. Discrete or qualitative data was summarized as proportions. All statistical analyses were conducted at a 95% significance level.

## Results

In our study, gender distribution was almost equal with females constituting 53.3% (n=16) of the sample, and the rest 46.7% (n=14) were males. Out of 30 subjects enrolled, 40% (n=12) of cases were in the age group of 51-60 years, followed by 33.3% (n=10) cases in 61-70 years, 16.7% (n=5) in 41-50 years, and 10.0% (n=3) in the 71-80 years age group. The mean age of cases was 58.77±9.01 years. 

Kujala scores, knee ROM, and PTS angle were taken at 3 different time points: pre-op, post-op day 7, and 3 months post-op (Table 1).

**Table 1:** Mean values of variables at different time points ROM: range of motion; PTS: posterior tibial slope

Variables	Preoperative	Postoperative Day 7	Postoperative (3 Months)
	(Mean±SD)		
Kujala Score	39.43±7.51	54.27±9.71	73.4±8.1
Knee ROM	66.50±14.81	88.67±15.81	114.0±6.62
PTS Angle	9.69±5.82	5.3±2.84	5.3±2.84

**Figure 3:**
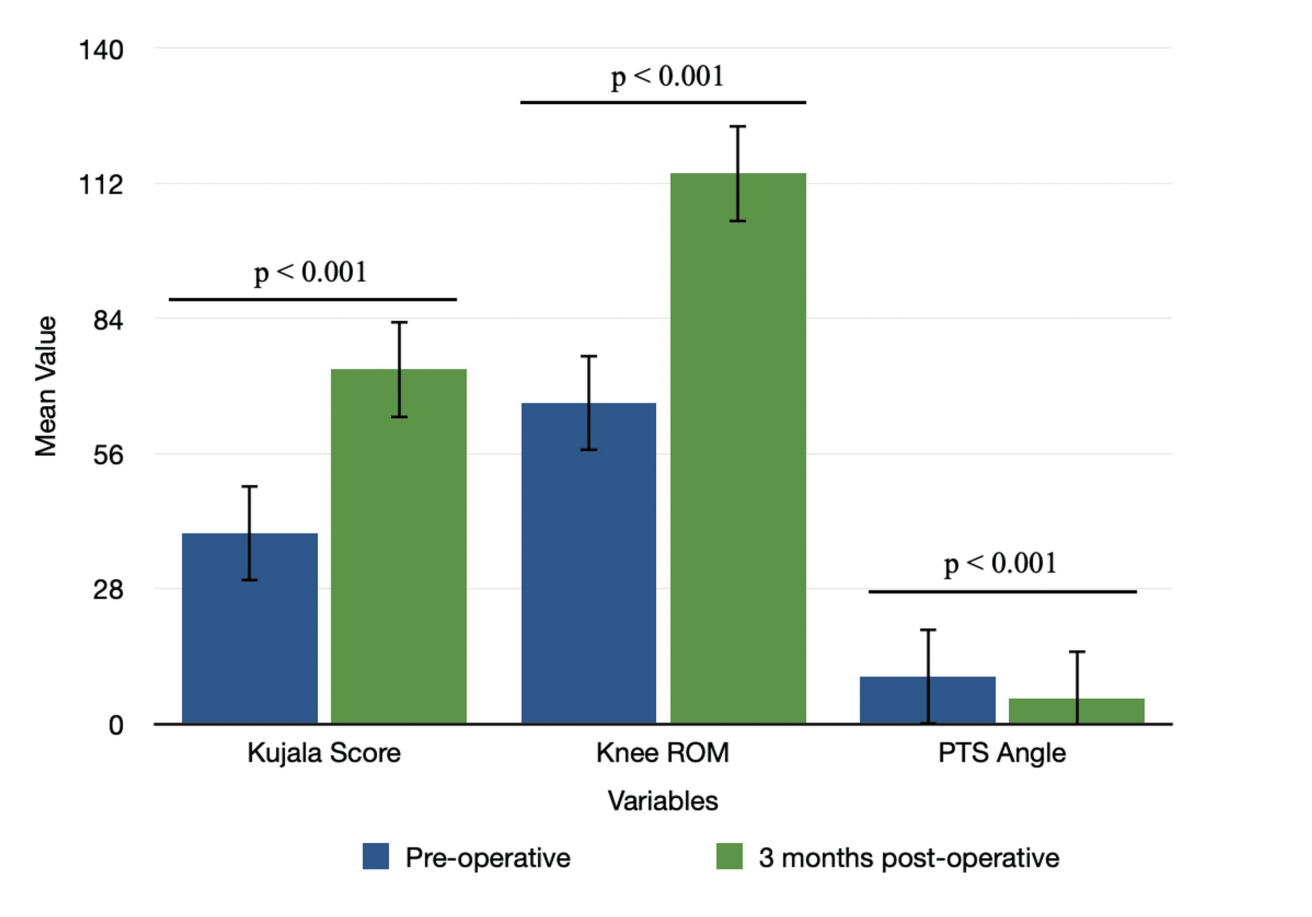
Mean change in Kujala score, knee ROM, and PTS angle from preoperative to postoperative three months ROM: range of motion; PTS: posterior tibial slope

In our study, the mean Kujala score, and knee ROM increased significantly during post-op day 7 and after 3 months when compared to pre-op status (p<0.001) while the mean PTS angle was significantly decreased (p<0.01). Conversely, no significant changes in PTS angle were observed after 3 months, compared to post-op day 7 (Table 2 and Figure 3). 

**Table 2:** Change in the mean value of variables from preoperative to three months postoperative *paired t-test was used for statistical analysis ROM: range of motion; PTS: posterior tibial slope

Variable	Preoperative	Postop (3 Months)	t-value, Df	p-value
Kujala Score	39.43±7.51	73.4±8.1	21.994, 29	<0.001
Knee ROM	66.5±14.81	114±6.62	18.743, 29	<0.001
PTS Angle	9.69±5.82	5.3±2.84	3.858, 29	<0.001

There was no significant difference found in the mean value of Kujala score, knee ROM, and PTS angle between males and females (Table 3).

**Table 3:** Difference in the mean value of variables between male and female *student’s t-test was used for statistical analysis ROM: range of motion; PTS: posterior tibial slope

Variable		Male	Female	t value, DF	p-value
Preoperative	Kujala Score	38.64±7.28	40.13±7.88	0.535, 28	0.599
	Knee ROM	67.14±11.39	65.94±17.63	0.218, 28	0.828
	PTS Angle	9.36±6.71	9.98±5.13	0.286, 28	0.778
Postoperative	Kujala Score	55.57±10.65	53.13±9	0.680, 28	0.501
	Knee ROM	87.5±19.88	89.69±11.76	0.373, 28	0.712
	PTS Angle	5.57±2.7	5.07±3.02	0.475, 28	0.636
Postoperative (3 months)	Kujala Score	73.14±7.55	73.63±8.79	0.163, 28	0.874
	Knee ROM	112.5±8.03	115.31±4.99	1.167, 28	0.252
	PTS Angle	5.57±2.7	5.07±3.02	0.475, 28	0.636

Among cases with < 4 degrees, 4-8 degrees, and >8 degrees change in PTS angle, the mean change in Kujala score was 15.24±6.59, 14.83±6.68, and 13.86±2.19 respectively. The association of change in PTS angle with change in Kujala score from pre-op to post-op day 7 was found to be statistically insignificant. Similarly, the association of change in PTS angle with change in knee ROM was found to be statistically insignificant (p=0.875).

The correlation of change in PTS angle with change in Kujala score and change in knee ROM was statistically insignificant (Table 4).

**Table 4:** Correlation of change in the PTS angle with the Kujala score and knee ROM ROM: range of motion; PTS: posterior tibial slope

Variable	Spearman's Correlation Coefficient	p-Value
Change in PTS - Kujala (Preop to Postop)	-0.173	0.361
Change in PTS - Kujala (Preop to 3 months)	-0.055	0.773
Change in PTS - Knee ROM (Preop to Postop)	-0.066	0.729
Change in PTS - Knee ROM (Preop to 3 months)	-0.012	0.951

The mean Kujala score among cases with a PTS angle <4 was 50.91±13.06, and the Kujala score was higher in cases with a higher PTS angle. The association of Kujala score with PTS angle was found to be statistically insignificant. Similarly, the mean value of Knee ROM was higher in cases with increased PTS angle, and the association of PTS angle with knee ROM was also insignificant.

## Discussion

In the present study, 30 patients were included with a mean age of 58.77±9.01 years, comparing the demographic distribution of patients across several studies, including those by Elsheikh 2019 [15], Kastner et al. 2014 [6], Adıyeke et al. 2022 [2], and Seo et al. 2013 [19]; statistically insignificant observations emerged.

In a study by Kastner et al., 66 patients were investigated with the average patient age at the time of surgery being 76±11.97 years (range 21-78 years) [6]. The Elsheikh study included a total of 60 patients with a mean age of 66.87±5.37 years [15]. In another study by Adıyeke et al., a total of 64 patients were included with a mean age of 68±9.2 years [2]. Seo et al., in their study, enrolled patients with a mean age of 68.4±8.1 years [19]. From the total number of procedures, 436 were performed on the right knee while 465 were carried out on the left knee. The study population consisted of 134 male patients and 677 female patients.

In the present study, gender distribution was almost equal, with females constituting 53.3% of the sample and the rest being males. In the present study, a statistically insignificant association (p=0.778) was recorded between gender and PTS angle similarly. In a study by Kastner et al., out of 66 patients, 50 were women (76%) and 16 men (24%) included. In the study by Elsheikh, out of 60 patients, 37 were women (62%) and 23 men (38%) were included [15]. Adıyeke et al. conducted a study on knee observations from a cohort of 64 patients, composed of 10 males and 54 females [2]. Seo et al. 2013 included 134 (16.7%) males and 677 (83.3%) females in their study [19]. There was no statistically significant difference in the demographic data of the mentioned studies.

In the present study, knee ROM changes significantly from preop to postop day 7 but no significant relation was found between change in PTS and change in knee ROM (p=0.295). Assessing functional outcomes in previous studies, such as Adıyeke et al. [2], no statistically significant relationship was found between knee ROM during the final follow-up between different groups based on the posterior tibial slope (PTS).

Kastner et al. investigated the effect of PTS on ROM after low-contact motion-bearing TKA and found no relationship between tibial inclination and ROM [6]. In a study by Seo et al., knee ROM was improved postoperatively in all groups, but the level of improvement was not significantly different among the groups (p=0.241) [19].

A cadaveric study by Agneskirchner et al. found that a gradual increase in tibial inclination up to 10 degrees produced a gradual increase in the degree of flexion up to 10.6 degrees [13].

In our study, the Kujala score showed a significant increase from preop to postop with scores of 54.27±9.71 and 73.4±8.1, respectively (p<0.001). In a previous study by Adıyeke et al., the Kujala score was 53.15±5.5 in group I patients (with a PTS angle of <50) and 75.1±5.5 in group II (with PTS angle of >100) and no significant difference between the mean Kujala scores among the two groups (p=0.026) [2].

In the present study, an association of change in the PTS angle with change in the Kujala score from preop to postop was found to be statistically insignificant. In a study by Seo et al., patients were divided into 5 groups, according to the change in PTS that was calculated by subtracting the preop from the postop PTS: group 1, >30; group 2, 30 to 10; group 3, 10 to -10; group 4, -10 to -30; and group 5, <- 30 [19]. The Kujala score was increased postoperatively in all groups, and especially significantly in group 2 (76.5, p=0.041) and group 3 (77.4, p=0.029).

The groups categorized based on alterations in preoperative and postoperative PTS exhibited no statistically significant variations in the changes of knee ROM and Kujala score before and after surgery. We concluded that this was due to the prevalence of degenerative arthritis in most patients, and the fact that PTS is not the sole factor influencing post-surgical pain, limitations in ROM, and knee functionality. As this research was conducted at a single center, inherent biases and limitations exist in patient selection, surgical approaches, postoperative management protocols, and follow-up duration, potentially affecting the study's external validity and reproducibility.

## Conclusions

Initial evaluations revealed compromised knee functionality, evidenced by reduced Kujala scores and restricted knee ROM, associated with higher PTS angles. Following surgical intervention, knee function and mobility showed marked improvement, as indicated by enhanced Kujala scores and increased knee ROM. A notable decrease in the mean PTS angle was observed postoperatively, which inversely correlated with knee ROM. Nevertheless, alterations in the PTS angle did not demonstrate a significant relationship with changes in Kujala scores. The positive outcomes were maintained over three months, suggesting medium-term effectiveness, with no substantial differences observed between sexes, indicating comparable efficacy of the intervention across genders.

The study concludes that PTS does not significantly influence functional outcomes, particularly knee ROM, in patients undergoing primary TKA. Subsequent research should encompass multicenter studies with larger cohorts and extended follow-up periods to further explore the relationship between PTS and knee ROM following TKA.
